# Estimated public health impact of concurrent mask mandate and vaccinate-or-test requirement in Illinois, October to December 2021

**DOI:** 10.1186/s12889-024-18203-8

**Published:** 2024-04-12

**Authors:** François M. Castonguay, Arti Barnes, Seonghye Jeon, Jane Fornoff, Bishwa B. Adhikari, Leah S. Fischer, Bradford Greening, Adebola O. Hassan, Emily B. Kahn, Gloria J. Kang, Judy Kauerauf, Sarah Patrick, Sameer Vohra, Martin I. Meltzer

**Affiliations:** 1 National Center for Emerging and Zoonotic Infectious Diseases, Division of Preparedness and Emerging Infections, Centers for Disease Control and Prevention, Health Economics and Modeling Unit, U.S. Department of Health and Human Services, 7101 Avenue du Parc, Local 3180, QC H3N 1X9 Atlanta, Georgia; 2 Contact Tracing and Innovation Section (CTIS), State Tribal Local and Territorial (STLT) Task Force, CDC COVID-19 Response; Centers for Disease Control and Prevention, Modeling Support Team, U.S. Department of Health and Human Services, Atlanta, Georgia; 3 Illinois Department of Public Health, Springfield, IL USA; 4 Department of Health Management, Evaluation and Policy, University of Montreal School of Public Health, and Centre for Public Health Research – CReSP, 7101 Av du Parc, 3E Étage, Montréal, QC H3N 1X9 Canada

**Keywords:** Masks, Vaccines, Cases and hospitalizations averted, COVID-19, Modeling

## Abstract

**Background:**

Facing a surge of COVID-19 cases in late August 2021, the U.S. state of Illinois re-enacted its COVID-19 mask mandate for the general public and issued a requirement for workers in certain professions to be vaccinated against COVID-19 or undergo weekly testing. The mask mandate required any individual, regardless of their vaccination status, to wear a well-fitting mask in an indoor setting.

**Methods:**

We used Illinois Department of Public Health’s COVID-19 confirmed case and vaccination data and investigated scenarios where masking and vaccination would have been reduced to mimic what would have happened had the mask mandate or vaccine requirement not been put in place. The study examined a range of potential reductions in masking and vaccination mimicking potential scenarios had the mask mandate or vaccine requirement not been enacted. We estimated COVID-19 cases and hospitalizations averted by changes in masking and vaccination during the period covering October 20 to December 20, 2021.

**Results:**

We find that the announcement and implementation of a mask mandate are likely to correlate with a strong protective effect at reducing COVID-19 burden and the announcement of a vaccinate-or-test requirement among frontline professionals is likely to correlate with a more modest protective effect at reducing COVID-19 burden. In our most conservative scenario, we estimated that from the period of October 20 to December 20, 2021, the mask mandate likely prevented approximately 58,000 cases and 1,175 hospitalizations, while the vaccinate-or-test requirement may have prevented at most approximately 24,000 cases and 475 hospitalizations.

**Conclusion:**

Our results indicate that mask mandates and vaccine-or-test requirements are vital in mitigating the burden of COVID-19 during surges of the virus.

**Supplementary Information:**

The online version contains supplementary material available at 10.1186/s12889-024-18203-8.

## Introduction

Public health mandates or requirements are laws put into place to promote healthy behaviors that mitigate disease burden. The mechanisms through which these policies interact with public health can vary substantially. For instance, a mask mandate aims to reduce disease transmission and a vaccinate-or-test requirement aims to reduce exposure to infectious cases through testing and also reduce the incidence of severe disease and mortality through vaccination. Several studies have estimated the impact of face mask mandates [1–4] and vaccination requirements [5, 6] on COVID-19 transmission.

Facing a surge in COVID-19 cases during the fall of 2021 due to the emergence of the Delta variant [7], the Governor of Illinois issued an executive order on August 26, 2021 [8] that required workers in certain frontline professions (*e.g.*, healthcare workers, school personnel, higher education personnel, and state owned or operated congregate facilities) to get vaccinated against COVID-19 (i.e., at least receive the first dose of a two-dose COVID-19 vaccine series), or undergo, at a minimum, weekly testing for COVID-19 if they remained unvaccinated by September 19, 2021 [9]. At the latest, individuals had to receive their second dose of a two-dose COVID-19 vaccine series 30 days after the September 19^th^ deadline [9]. There was also a mandate that required face mask use for any individual in a public indoor setting, beginning August 30, 2021, regardless of vaccination status. The August 26, 2021 executive order [8] was issued while the rate of cases continued to rise steeply, despite an earlier mask mandate for schools, daycares, and long-term care facilities enacted on August 4, 2021 [10]. The Delta variant represented more than 99% of sampled strains in Illinois at that point [7] and healthcare capacity was strained.

In this paper, we provide estimates of cases and hospitalizations averted by the concomitant mask mandate and vaccinate-or-test requirement in Illinois between October 20 and December 20, 2021. We estimate separately the impact of increases in masking and vaccination. We adjust for a wide range of compliance levels by estimating the impact for various levels of mask-wearing pre- and post-mandate, and for various potential levels of vaccination uptake in the front-line workforce that would follow the announcement of the vaccinate-or-test requirement.

## Methods

We estimated the impact of increases in masking and vaccination on COVID-19 incidence in Illinois from October 20 to December 20, 2021. Previous reports have shown that use of face masks reduces SARS-CoV-2 transmission [11–13]. We simplified estimating the impact of mask wearing, hereby referred to as mask effectiveness, by using (i) an average estimate of mask efficacy and (ii) an average percent of population compliant with correct mask wearing (see Technical Appendix for more details).^1^ For vaccines, the effectiveness of the vaccinate-or-test requirement depends on the difference between the percentage of population that complied with the executive order to vaccinate-or-test, and the percentage that were already vaccinated. This research activity was reviewed by the Centers for Disease Control and Prevention (CDC) and was conducted consistent with applicable federal law and CDC policy.^2^

We used CDC’s COVIDTracer modeling tool [14] to build an epidemic curve that mimicked the observed one in Illinois over the two-month period (October 20 - December 20, 2021).^3^ Similar to other studies [15–18] (Castonguay FM, Borah BF, Jeon S, Rainisch G, Kelso P, Adhikari BB, Daltry DJ, Fischer LS, Greening Jr B, Kahn EB, Kahn GJ, Meltzer MI: The Public Health Impact of COVID-19 Variants of Concern on the Effectiveness of Contact Tracing in Vermont, United States, unpublished), the two-month duration balances the need for sufficient time to pass after the start of the mandate to allow for an adequate assessment of the impact of the interventions being studied. Simultaneously, it aims to limit the potential for unknown confounding factors that may alter the impact of the interventions. We assumed that the effectiveness of interventions remained constant over the two-month study period. Those vaccinated following the vaccinate-or-test requirement should have achieved full vaccine-induced immunity^4^ by mid-October 2021, which matches the start of our analytic timeframe.^5^ COVIDTracer uses a compartmental Susceptible–Exposed–Infectious–Recovered (SEIR) mathematical model [19]. A user enters location-specific COVID-19 case counts, vaccination levels, a set of parameters describing COVID-19 epidemiology (*e.g.*, basic reproduction number), and estimates of the effectiveness of the interventions (Table 1) (see Technical Appendix for details and Appendix Table﻿ A4 for a list of Illinois-specific inputs).

**Table 1:** Input table for the COVID-19 epidemiological characteristics, mask mandate, and vaccinate-or-test requirement

Study Period (Dates covered)	Oct. 20 – Dec. 20, 2021
**Assumed COVID-19 Epidemiological Characteristics**	
Latent period duration^a^	2 days
Basic reproduction number^b^	R_0_ = 5
% cases asymptomatic^c^	40%
Infectiousness of asymptomatic cases^d^	75%
% vaccinated in general population^e^	53.8%
Vaccine effectiveness^f^	88%
Immunity duration^g^	180 days
**Inputs Specific to COVID-19 Mask Mandate**	
Mask effectiveness^h^	
Pre-mandate	3.6% – 16.8%
Post-mandate	6.1% – 21.3%
**Inputs Specific to COVID-19 Vaccinate-or-Test Requirement**	
Size of the population affected by requirement^i^	929,370
% vaccinated in sub-population affected by requirement^j^	
Pre-requirement	64.8%
Post-requirement	75.9%

^a^Per the literature, we used a two-day latent period associated with the SARS-CoV-2 Delta variant [20, 21]

^b^Per the literature, we used a basic reproduction number of R_0_ = 5.0 associated with the SARS-CoV-2 Delta variant [22, 23]. The infectivity distribution varies over time and is spread over an 11-day period [24, 25], and the effective reproduction number further depends on the estimated impact of non-pharmaceutical interventions (NPIs) and the size of the susceptible population, see Technical Appendix for more details

^c^Asymptomatic COVID-19 cases. Patients can be infected, and become infectious, without being symptomatic. They can and likely do contribute to onward transmission of the pathogen [26]

^d^Infectiousness of asymptomatic COVID-19 cases relative to symptomatic cases [26]

^e^Based on CDC COVID-19 vaccination data [27]—these data represent overall coverage among all ages at the beginning of the study period

^f^COVID-19 vaccine effectiveness is based on the effectiveness of two doses of the monovalent mRNA BNT162b2 (Pfizer-BioNTech, Comirnaty) against the Delta variant [28]

^g^We assume SARS-CoV-2 immunity lasts 180 days on average [29], this includes both vaccine-induced and disease-induced immunity

^h^Mask effectiveness is the product of (i) mask efficacy and (ii) mask compliance. The resultant values are the average mask effectiveness in the state of Illinois, for the time-period studied. See Technical Appendix for more details

^i^Based on Data from the U.S. Bureau of Labor Statistics [30]. See Appendix Table A2

^j^Based on data from the Centers for Disease Control and Prevention (CDC)’s National Healthcare Safety Network (NHSN) for CMS-certified nursing homes in IL [31]. Note that this data only represents a sub-population of the total population affected by the vaccinate-or-test requirement. See Appendix Table﻿ A3

### Impact of mask mandate

To model the impact of mandate-induced increased mask effectiveness, we first inputted a selected value from the range provided in Table 1 into COVIDTracer. The pre-mandate mask effectiveness range was 3.6%–16.8% and post-mandate mask effectiveness range of 6.1%–23.3%. For baseline analysis, we used post-mandate mask effectiveness of 14.2%, assuming 20% mask efficacy and 71% compliance. As it is very difficult to measure the degree of compliance with effective mask wearing in any population, we therefore constructed 24 scenarios of combinations of pre-and post-mandate mask effectiveness. We avoided over-estimating the impact of the mask mandate by using pre-mandate mask effectiveness values of less than 20% and only one post-mandate mask effectiveness value of over 20% (see Sensitivity Analyses).

We then “fitted” the curve of cumulative cases modeled by COVIDTracer to the jurisdiction’s reported cases by altering the percentage reduction in transmission ascribed to vaccine and various Non-Pharmaceutical Interventions (NPIs). The estimated percentage reduction in transmission that minimized the difference (*i.e.,* minimized the mean squared error) between the fitted and reported cumulative case curves is the estimate of the effectiveness of non-CICT NPIs (see Appendix for further details). Note that, because there were no measurements of the degree of under-reporting, we had to use the reported cases without any adjustments for possible under-reporting. Correcting for under-reporting may well have increased the estimates of cases and hospitalizations averted, for both mandates. Finally, we simulated what would have occurred without the mask mandate by re-setting the impact of mask effectiveness to one of 2 pre-mandate effectiveness levels (7.2% and 11.2%). The resulting plots are the number of cases that would have occurred without the mask mandate. The difference between the hypothetical plots of cases without mandate-induced increases in mask effectiveness and the plot of the reported cases (which includes the impact of mask mandate) are the cases averted due to the mask mandate. By “fitting the curve” (finding the best match between the SEIR model and the observed data), this methodology prevents over- or under-estimating the combined impact of all interventions.

#### Sensitivity analyses: mask mandate

Mask wearing compliance depends on several locality factors [32, 33]. As noted earlier, it is very difficult to measure the degree of compliance in large populations. We therefore constructed 24 scenarios of combinations of pre-and post-mandate mask effectiveness (Appendix Table﻿ A1).

### Impact of vaccinate-or-test requirement

To estimate the impact of the vaccinate-or-test requirement, we followed the same process as described above for masking (Table 1; see the Appendix for further details). The number of cases averted by the vaccinate-or-test requirement depended on (i) the baseline, pre-requirement, vaccination coverage, and (ii) the increase in vaccination coverage attributable to the requirement. We obtained an estimate of 929,370 frontline workers in Illinois who were potentially affected by the vaccinate-or-test requirement (see Appendix Table﻿ A2). The National Healthcare Safety Network (NHSN) reported that, for the period analyzed, vaccination coverage of the staff working in certified Centers for Medicare & Medicaid Services (CMS-certified) nursing homes in Illinois increased from 64.8% to 75.9%––an 11.1 percentage point increase in coverage (see Appendix Table﻿ A3). We used this 11.1 percentage point increase (equivalent to 103,160 additional persons vaccinated) as a base case scenario for analyzing the impact of the vaccinate-or-test requirement.

**Table 2:** Estimated cases and hospitalizations averted due to mask mandate for various levels of mask effectiveness^a^

Mask Effectiveness, Pre-mandate^b^	Mask Effectiveness, Post-mandate	Number of Cases Averted (Number of Hospitalizations Averted^c^)
3.6%	6.1%	149,817 (3,028)
5.0%		58,233 (1,177)
3.6%	7.1%	232,791 (4,705)
5.6%		83,250 (1,683)
7.2%	12.2%	415,085 (8,390)
10.0%		139,155 (2,813)
7.2%	14.2%	734,649 (14,849)
11.2%		211,125 (4,267)
10.8%	18.3%	901,144 (18,214)
15.0%		255,426 (5,163)
10.8%	21.3%	1,820,764 (36,801)
16.8%		417,605 (8,441)

^a^See Appendix Table﻿ A1 for the Results from all 24 scenarios of combinations of pre-and post-mandate mask effectiveness

^b^Assumes the levels of mask-wearing would have remained the same in the absence of the mask mandate

^c^Number of hospitalizations averted is calculated by multiplying the estimated number of averted cases by the infection-to-hospitalization ratio, which was assumed to be approximately 2.02% during that period [26]. Infection-to-hospitalization ratio is assumed not to vary with the assumed level of mask efficacy

**Table 3:** Estimated cases and hospitalizations averted by the vaccinate-or-test requirement for different increases in vaccination uptake

Percentage point increase in vaccination between 8/22 and 10/17 in population due to the vaccinate-or-test requirement (number of people this percentage represents)^a^		Number Averted
5.6 percentage point (51,580)	Cases	11,571
	Hospitalizations^b^	234
11.1 percentage point (103,160)	Cases	23,593
	Hospitalizations^b^	477

^a^Note that we do not know what percentage point can be attributed to the vaccinate-or-test requirement, so the two scenarios here represent examples meant to illustrate what could have happened

^b^Number of COVID-19 hospitalizations averted is calculated by multiplying the estimated number of averted cases by the infection-to-hospitalization ratio (factor of approximately 2.02%) [26]. Infection-to-hospitalization ratio is assumed not to vary with the vaccination coverage

#### Sensitivity analyses: vaccinate-or-test requirement

We do not know what proportion of the 11.1 percentage point increased coverage was due to the vaccinate-or-test requirement. Other factors, such as intent to be vaccinated regardless of the requirement or other requirements/mandates (*e.g.*, the federal mandate for CMS facilities announced during the study period), could have contributed to the increase. To address this uncertainty, we evaluated the impact of an arbitrary assumption that only half of the recorded increase in vaccine coverage could be attributable to the mandate (i.e., a 5.6 percentage point increase, equivalent to 51,580 additional persons vaccinated). Note that the potential indirect impact that the vaccinate-or-test requirement could have had on the general population [6] is not accounted for, which may have increased the overall impact of the requirement.

## Results

### Impact of mask mandate

**Fig. 1:**
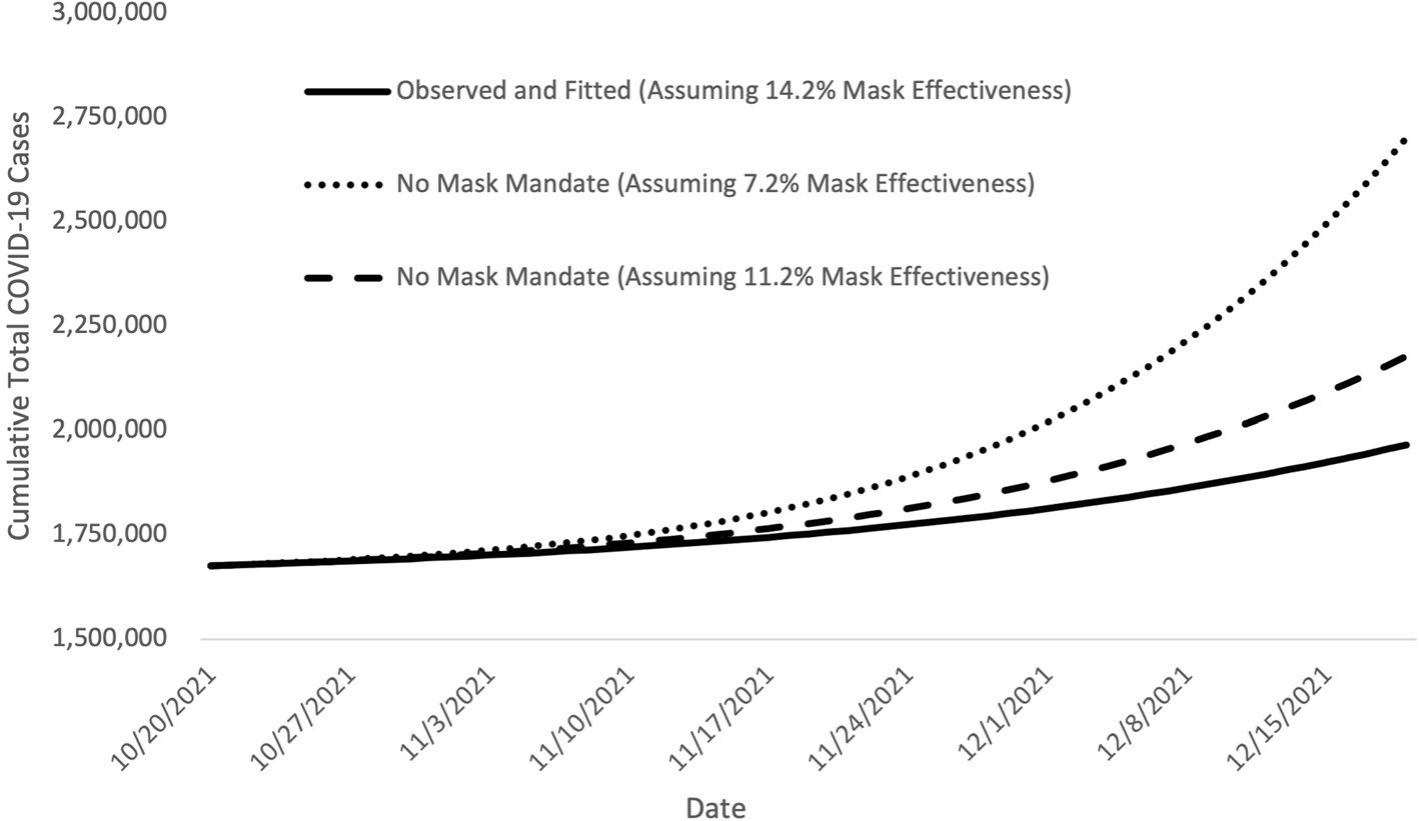
Fitted epidemic curves of COVID-19 case counts showing the impact of the mask mandate Notes﻿: Fitted epidemic curve of observed COVID-19 case counts, assuming a post-mandate mask effectiveness of 14.2%, and simulated epidemic curves assuming no mandate and continuation of pre-mandate mask effectiveness of either 7.2% or 11.2% (all three for the October 20 – December 20, 2021 period). The solid line is Illinois’s observed (fitted) cumulative COVID-19 case counts, and the broken lines are the simulated curves illustrating the cumulative total COVID-19 cases for the various scenarios that might have occurred if the mask mandate had not been enacted and mask efficacy was 20%. The differences between the solid and broken lines show the benefits of the mask mandate with greater divergence between the solid and broken lines indicating a greater impact. All results assume that the effects of nonpharmaceutical interventions —including masks—were constant over the two months shown

In Fig. 1, we present the plot of the cases assuming the post-mandate mask effectiveness of 14.2% (calculated assuming 20% mask efficacy and 71% compliance; represented by the solid black line in Fig. 1). This plot is then compared to the hypothetical plots of increased cases, assuming no mask mandate and a continuation of pre-mask effectiveness of either 7.2% or 11.2% (dotted and dashed lines). The cumulative difference between the dotted or dashed plotted lines and the solid line plot is the estimate of additional cases averted due to the mask mandate.

#### Sensitivity analyses: mask mandate

To calculate a lower-bound estimate of cases averted, we assumed that the mask mandate increased mask effectiveness from 3.6% to 6.1%. This resulted in an estimate of 149,817 additional cases and 3,028 additional hospitalizations averted due to the mask mandate (Table﻿ 2). We calculated an upper-bound estimate by increasing the mask effectiveness for pre- and post-mandate to 10.2% and 21.3%, respectively. This resulted in an upper estimate of 1,820,764 additional cases and 36,801 additional hospitalizations averted due to the mask mandate (Table 2). The remainder of the results in Table 1 show the estimates of cases and hospitalizations averted from another 10 scenarios. The results from all 24 scenarios of combinations of pre-and post-mandate mask effectiveness are presented in Appendix Table﻿ A1.

### Impact of vaccinate-or-test requirement

**Fig. 2:**
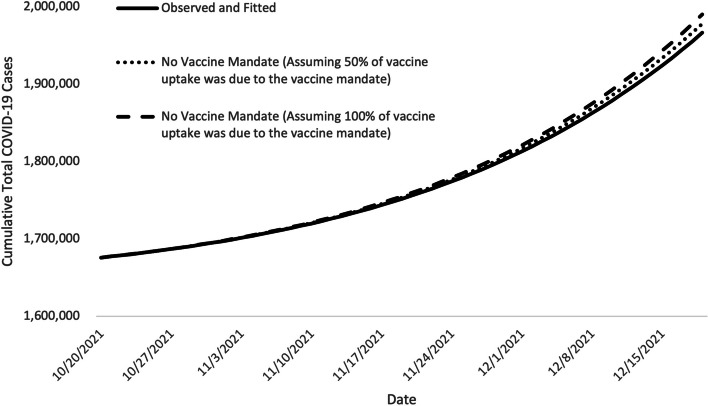
﻿Fitted epidemic curves of COVID-19 case counts showing the impact of the vaccinate-or-test requirement Notes﻿: Fitted epidemic curve of observed COVID-19 case counts and of two assumed increases in vaccination coverage attributable to the announcement of the vaccinate-or-test requirement (for October 20 – December 20, 2021). These represent approximately 51,580 and 103,160 individuals vaccinated because of the vaccinate-or-test requirement. The solid line is Illinois’s observed cumulative COVID-19 case counts, and the dashed and dotted lines are the simulated curves illustrating the cumulative total cases for scenarios where there would have been a lower vaccine uptake without the vaccinate-or-test requirement. The differences between the solid and dashed or dotted lines show the number of cases averted by the vaccinate-or-test requirement. All results assume that the effects of other nonpharmaceutical interventions (NPIs) were constant over the two months analyzed

Assuming that the vaccinate-or-test requirement resulted in an 11.1 percentage point increase in persons vaccinated, we estimated that 23,593 cases and 477 hospitalizations were averted (Table﻿ 3 and Fig. 2).

#### Sensitivity analyses: Vaccinate-or-test Requirement

When we assumed that only half of the increase was attributable to the vaccinate-or-test requirement, an estimated 11,571 cases and 234 hospitalizations were averted by the vaccinate-or-test requirement (Table 3 and Fig. 2).

## Discussion

We estimated that increases in masking following the announcement of the mask mandate may have averted at least 58,000 cases in Illinois. The vaccinate-or-test requirement among frontline workers averted up to 24,000 cases during the period studied. The assumed post-mandate mask effectiveness (6.1% - 21.1%) was the most influential variable assessing the impact of mask mandates.

Our results provide data-driven evidence that can inform decision-making regarding public health interventions during times of surge. While there have been concerns regarding the negative impact of such mitigation measures [34], mask mandates are impactful [35] and vaccination requirements have demonstrated the ability to strengthen vaccination intentions across racial and ethnic groups, and even those who may be resistant [36]. Public adherence to these practices due to requirements as opposed to free choice is a more complicated debate. Several studies have demonstrated that while vaccine mandates may result in some vaccine hesitancy, they have been associated with improved vaccination rates [37, 38]. Hospital staff vaccination reports have found that many employees chose vaccination over resignation [37].

Our study has limitations. Several factors made it difficult to use a direct causal identification methodology, such as difference-in-differences. These factors included the absence of a credible control group and several confounding factors due to the important period between the announcement of the intervention and when compliance was required. We had to make several assumptions because the precise impact that the concomitant mask mandate and vaccinate-or-test requirement in Illinois had on COVID-19 burden depended largely on several unobserved factors, namely mask quality, level of mask-wearing pre- and post-mandate, and the proportion of vaccine uptake attributable to mandate. Further, while we estimated the impact of increases in masking and vaccination separately, there may have been unaccounted synergistic effects from combining both interventions [39]. We also assumed that the impact of face masks and vaccination remained constant over the period of study. To reduce the potential impact of such assumption, we limit our study period to two months. We do not account for partial immunity (*e.g.,* if an individual received their first vaccine shot during the study period), and hence assume individuals are either fully susceptible (because the individual was never vaccinated or never infected, or because immunity acquired through vaccination or prior infection was more than 180 days ago [29] and is no longer protective) or fully immune (due to prior infection or vaccination) during the two-month study period. By doing so, we may underestimate the impact of the policies (e.g., because the first dose of a two dose COVID-19 vaccine series may still provide some protection [40] which would increase the impact of the vaccinate-or-test requirement) or overestimate the impact of the policies (e.g., because those protected by the first dose of a two dose COVID-19 vaccine series would not have been protected by masks), with the overall direction of the bias being uncertain. There is also the possibility that the vaccinate-or-test requirement for frontline workers may have had an impact on the general population, as the requirement may have signaled the importance of vaccination for individuals not directly covered by the vaccinate-or-test requirement [6]. Finally, we assumed that there are no differences in disease transmission that are attributable to age, location, or occupation—in other words, every individual in the population is assumed to have the same risk of catching COVID-19 and is assumed to behave in the same way as any other individual. Those affected by the vaccinate-or-test requirement were frontline professionals who could have had potentially very different mixing patterns compared to the general population.

During the two-month study period, almost 2,000 hospitalizations were averted according to our model. Had these hospitalizations occurred, they would have had a significant impact on an already strained healthcare system. These findings can help control viral transmission of diseases other than COVID-19 at both hospital and community levels, and will help refine future decisions on the timing and scale of such public health measures should we find ourselves again in a similar healthcare crisis.

### Supplementary Information

**Supplementary Material 1. **

## Data Availability

No datasets were generated or analysed during the current study.

## Footnotes

1 Mask effectiveness is the product of (i) mask efficacy and (ii) mask compliance. Mask efficacy is defined as the extent to which masks reduce the output and uptake of the virus droplets/aerosols (range 10%-30%, see Ueki et al. [11]) and mask compliance is defined as the percentage of the population properly wearing masks.

2 See e.g., 45 C.F.R. part 46, 21 C.F.R. part 56; 42 U.S.C. §241(d); 5 U.S.C. §552a; 44 U.S.C. §3501 et seq.

3 This modeling tool has been used by several studies to estimate the impact of CICT [15–18] (Castonguay FM, Borah BF, Jeon S, Rainisch G, Kelso P, Adhikari BB, Daltry DJ, Fischer LS, Greening Jr B, Kahn EB, Kahn GJ, Meltzer MI: The Public Health Impact of COVID-19 Variants of Concern on the Effectiveness of Contact Tracing in Vermont, United States, unpublished) along with instructions provided for replicating this analysis and model use [15, 16].

4 We defined fully vaccinated as either having received two doses of the monovalent mRNA BNT162b2 (Pfizer-BioNTech, Comirnaty) or monovalent mRNA mRNA-1273 (Moderna, Spikevax) COVID-19 vaccine, or one dose of the single-dose adenovirus vector-based Ad26.COV.S (Janssen [Johnson & Johnson]) COVID-19 vaccine [48].

5 Recall that the vaccinate-or-test requirement required individuals to get their first dose of a two-dose COVID-19 vaccine series, or undergo, at a minimum, weekly testing for COVID-19 if they remained unvaccinated by September 19, 2021 [9]. CDC recommended at least three weeks between two doses for Pfizer-BioNTech and 28 days between two doses for Moderna any two vaccine [49]. To comply with the requirement, individuals had to receive their second dose of a two-dose COVID-19 vaccine series on October 19, 2021, at the latest.
