# Supplementary material for: Estimated public health impact of concurrent mask mandate and vaccinate-or-test requirement in Illinois, October to December 2021
Source: BMC Public Health. 2024 Apr 12;24:1013. doi: 10.1186/s12889-024-18203-8 (PMC11010411; doi:10.1186/s12889-024-18203-8)
Supplement: Supplementary file 1 — Supplementary Material 1. [file 12889_2024_18203_MOESM1_ESM.docx]

# Appendix

## Estimated Public Health Impact of Concurrent Mask Mandate and Vaccinate-or-Test Requirement in Illinois, October to December 2021

### BMC Public Health

François M. Castonguay^1,2,4*^, Arti Barnes^3^, Seonghye Jeon^1,2^, Jane Fornoff^3^, Bishwa B. Adhikari^1,2^, Leah S. Fischer^1,2^, Bradford Greening Jr.^1,2^, Adebola O. Hassan^3^, Emily B. Kahn^1,2^, Gloria J. Kang^1,2^, Judy Kauerauf^3^, Sarah Patrick^3^, Sameer Vohra^3^, Martin I. Meltzer^1,2^

^1^National Center for Emerging and Zoonotic Infectious Diseases, Division of Preparedness and Emerging Infections, Health Economics and Modeling Unit; Centers for Disease Control and Prevention, U.S. Department of Health and Human Services, Atlanta, GA.

^2^Modeling Support Team, Contact Tracing and Innovation Section (CTIS), State Tribal Local and Territorial (STLT) Task Force, CDC COVID-19 Response; Centers for Disease Control and Prevention, U.S. Department of Health and Human Services, Atlanta, GA.

^3^Illinois Department of Public Health, Springfield, IL.

^4^Department of Health Management, Evaluation and Policy, University of Montreal School of Public Health, and Centre for Public Health Research – CReSP; 7101 Av du Parc, 3e étage, Montréal, QC H3N 1X9, Canada.

**^*^Correspondence:**

François M. Castonguay

[francois.castonguay@umontreal.ca](mailto:francois.castonguay@umontreal.ca)

## Supplementary Tables

| \| **Appendix Table A1.** Estimated COVID-19 cases and hospitalizations averted by the increase in masking following the announcement of the mask mandate in Illinois for various levels of pre- and post-mandate mask effectiveness.   \| **Mask Effectiveness, Pre-mandate**^*^**^†^** \| **Mask Effectiveness, Post-mandate^†^** \| **Number of Cases Averted (Number of Hospitalizations Averted^‡^)** \| \| --- \| --- \| --- \| \| 3.6% \| 6.1% \| 149,817 (3,028) \| \| 3.8% \| 135,389 (2,736) \| \| 4.8% \| 70,037 (1,416) \| \| 5.0% \| 58,233 (1,177) \| \| 3.6% \| 7.1% \| 232,791 (4,705) \| \| 4.4% \| 166,939 (3,374) \| \| 4.8% \| 137,149 (2,772) \| \| 5.6% \| 83,250 (1,683) \| \| 7.2% \| 12.2% \| 415,085 (8,390) \| \| 7.6% \| 367,201 (7,422) \| \| 9.6% \| 170,884 (3,454) \| \| 10.0% \| 139,155 (2,813) \| \| 7.2% \| 14.2% \| 734,649 (14,849) \| \| 8.8% \| 483,015 (9,763) \| \| 9.6% \| 379,727 (7,675) \| \| 11.2% \| 211,125 (4,267) \| \| 10.8% \| 18.3% \| 901,144 (18,214) \| \| 11.4% \| 779,263 (15,750) \| \| 14.4% \| 321,451 (6,497) \| \| 15.0% \| 255,426 (5,163) \| \| 10.8% \| 21.3% \| 1,820,764 (36,801) \| \| 13.2% \| 1,107,929 (22,393) \| \| 14.4% \| 831,304 (16,802) \| \| 16.8% \| 417,605 (8,441) \| \| \| --- \| --- \| --- \| --- \| --- \| --- \| --- \| --- \| --- \| --- \| --- \| --- \| --- \| --- \| --- \| --- \| --- \| --- \| --- \| --- \| --- \| --- \| --- \| --- \| --- \| --- \| --- \| --- \| --- \| --- \| --- \| --- \| --- \| --- \| --- \| --- \| --- \| --- \| --- \| --- \| --- \| --- \| --- \| --- \| --- \| --- \| --- \| --- \| --- \| --- \| --- \| --- \| --- \| --- \| --- \| --- \| --- \| --- \| \| ^*^Assumes the levels of mask-wearing would have remained the same in the absence of the mask mandate.  ^†^Mask effectiveness is the product of (i) mask efficacy and (ii) mask compliance. Mask efficacy is defined as the extent to which masks reduce the output and uptake of the virus droplets/aerosols (range 10%-30%, see Ueki et al. [11]) and mask compliance is defined as the percentage of the population properly wearing masks. Mask compliance is not observed, so we provide a range for illustrative purposes (pre-mandate range: 36% ­– 56%; post-mandate range: 61% – 71%).  ^‡^Number of hospitalizations averted is calculated by multiplying the estimated number of averted cases by the infection-to-hospitalization ratio, which was approximately equal to 2.02% during that period [29]. Infection-to-hospitalization ratio is assumed not to vary with the assumed level of mask efficacy. \|   **Appendix Table A2.** Number of people in Illinois working in high-risk occupations that were affected by the vaccinate-or-test requirement. |
| --- | --- | --- | --- | --- | --- | --- | --- | --- | --- | --- | --- | --- | --- | --- | --- | --- | --- | --- | --- | --- | --- | --- | --- | --- | --- | --- | --- | --- | --- | --- | --- | --- | --- | --- | --- | --- | --- | --- | --- | --- | --- | --- | --- | --- | --- | --- | --- | --- | --- | --- | --- | --- | --- | --- | --- | --- | --- | --- | --- |
| \|  \| **Population** \| \| --- \| --- \| \| **Educational Instruction and Library Occupations** \| 353,970 \| \| **Healthcare Practitioners and Technical Occupations** \| 344,350 \| \| **Healthcare Support Occupations** \| 209,950 \| \| **Protective Service Occupations^*^** \| 21,100 \| \| **Total** \| **929,370** \| |
| **Notes.** These data are based on the U.S. Bureau of Labor Statistics, Occupational Employment and Wage Statistics, May 2020 State Occupational Employment and Wage Estimates [33]. These groups do not perfectly match the vaccination groups as defined in the executive order [9]—these numbers are meant to provide a rough estimate.  * This is based on estimates provided by the Illinois Department of Public Health. |
| **Appendix Table A3.** Vaccination levels pre- and post- vaccinate-or-test requirement in nursing homes in Illinois. |
| \|  \| **Vaccination levels by date in**  **CMS-certified nursing homes** \| \| **Estimate of number of workers** \| \| --- \| --- \| --- \| --- \| \|  \| 8/22/2021 \| 10/17/2021 \| – \| \| **Nursing Homes** \| 64.8% \| 75.9% (+11.1 pp.) \| 82,591 \| |
| **Notes.** These data are based on COVID-19 vaccination coverage and reporting among staff in nursing homes in Illinois, as reported by the National Healthcare Safety Network (NHSN) [34] and U.S. Bureau of Labor Statistics [33]. |

# Technical Appendix

We utilize the COVIDTracer Advanced modeling tool [14] developed by the U.S. Centers for Disease Control and Prevention (CDC); in the main paper we simply refer to this as “COVIDTracer.” This tool employs a compartmental SIR epidemiological model to mimic the COVID-19 dynamics (see [42] for a detailed discussion of compartmental models for COVID-19), which simulates the evolution in the number of susceptible ($S$), infected ($I$), and removed ($R$) individuals over time. It is designed to assess the public health impact of case investigation and contact tracing (CICT), as demonstrated in several aforementioned studies [15, 16, 17, 18, 19]. For this paper, we adapted the tool and incorporated Illinois-specific data to simulate hypothetical epidemic curves to explore scenarios in which masking or vaccination would have been reduced, mimicking the absence of mask mandate and vaccinate-or-test requirement (see Modeling Interventions section for more details). The difference between the observed case count and the modeled case counts under reduced masking or vaccination scenarios yields estimates of the impact of the mask mandate or vaccinate-or-test requirement. We assume that transmission reductions due to nonpharmaceutical interventions (NPIs)—including CICT and other NPIs such as masking—remain constant over the period of analysis (two months).

## Model of Disease Transmission

We use a susceptible­­–infected–removed (SIR) model to forecast the disease dynamics in Illinois. We assume the jurisdiction’s total population is closed in the sense that there is no external introduction of infected individuals, such as from neighboring states (see [43] for example), and that the jurisdiction’s population size remains constant over the period of analysis (thus excluding births and deaths from our model). Given the short time-horizon of our analysis (two months), we assume immunity resulting from vaccination remains constant over the entire analytical period. This simplifies the model-fitting process, as we do not have to account for complex vaccination dynamics.

In the absence of NPIs, the change in susceptible individuals between any two days is

$$\dot{S}=-\sum_{i=1}^{n} \beta_{i}S\frac{I_{i}}{N}$$

where $\beta_{i}$ is the effective contact rate of individuals that were infected $i$ days ago ($n$ is the duration of infection, in days), $S$ is the number of susceptible individuals, $I_{i}$ is the number of infected individuals that were infected $i$ days ago, $N$ is the total size of the jurisdiction’s population. An implication arising from this equation is that we do not account for age- or location-based heterogeneities in transmission; in other words, we assume homogeneous mixing among individuals.

Once infected, individuals transition into the infected class $I_{1}$, where the change in individuals infected $i=1$ day ago is

$$\dot{I}_{1}=\sum_{i=1}^{n} \beta_{i}S\frac{I_{i}}{N}-\gamma I_{1}$$

and the change in individuals infected $i=2, \ldots, n$ days ago is

$$\dot{I}_{i}=\gamma I_{i-1}-\gamma I_{i}$$

where $\gamma$ is the duration an individual spends in each infectious compartment (*i.e.,* one day). Note that we implicitly account for an exposed (E) class in the above infectious compartments by setting $\beta_{i}=0$ for $i=1,\ldots, \mathcal{l}$, where $\mathcal{l<}n$ is the length (in days) of the latent period.

At the beginning of the analysis, the jurisdiction’s population that is fully protected (*i.e.,* the “removed” individuals) is defined as the sum of (i) the individuals who have been fully and successfully vaccinated within six months prior to the analysis’s start date (thus accounting for potential vaccine failure and potential reinfection of individuals vaccinated in the six months prior to the analysis’s start date) and (ii) the individuals who have been infected within the last six months (regardless of whether they were vaccinated). There are a few implicit assumptions that are embedded in this calculation. First, we assume that both naturally acquired and vaccine-acquired immunity last for 180 days [32] and offer the same level of protection. Second, the susceptibility to infection is the same for individuals that were never vaccinated and for individuals who were vaccinated more than six months ago. Third, the likelihood of receiving vaccination is the same regardless of prior infection status. Fourth, we assume no partial immunity—individuals are either fully protected or fully susceptible. Furthermore, individuals previously infected and vaccinated remain protected throughout the two-month analysis period; any potential waning immunity effects within this timeframe are disregarded. As a result, the number of removed individuals in the jurisdiction’s population changes according to

$$\dot{R}=\gamma I_{n}$$

since immunity through vaccination is assumed constant over the period analyzed.

## Modeling Interventions

The above disease transmission model can be modified to account for transmission reductions due to NPIs. Non-CICT NPIs like masking lead to a reduction of all contact rates $\beta_{i}$ by a certain proportion. CICT entails making some $\beta_{i}$’s equal to zero because of isolation or quarantine of infected individuals. Using the above notation, the basic reproduction number, $R_{0}$, of this model is

$R_{0}=\frac{\sum_{i=1}^{n} \beta_{i}}{\gamma}$.

The model can disentangle the public health impact of the CICT program from the public health impact of all other NPIs. The reduction in transmission attributed to the CICT program (*i.e.,* CICT effectiveness) were computed by integrating two crucial jurisdiction-specific “performance values” into the model: (i) the proportion of cases and contacts that entered isolation and quarantine, and (ii) the days required to do so (see Appendix Table A4 for these values). We assume levels of compliance with isolation and quarantine guidelines (see Appendix Table A5 for these values), and we use the SIR model described above to obtain an estimate of the number of COVID-19 cases averted by CICT by comparing the scenarios with and without the CICT program. In cases where the CICT program is absent, we assume that solely cases and contacts who entered isolation and quarantine are those who did so voluntarily. This way of calculating the public health impact of the CICT program implicitly implies that some cases and contacts would only go into isolation or quarantine if they had been interviewed or notified by the CICT program, and that the effect of the CICT program on the reduction in disease transmission is constant over the entire study period (two months).

| **Appendix Table A4.** COVID-19 burden, observed case investigation and contact tracing (CICT) program metrics, and calculated CICT effectiveness in Illinois. | |  |
| --- | --- | --- |
| **Evaluation Date**  **COVID-19 Burden**  Mean daily cases^*^  Reported Cases  **% Population Fully Vaccinated** | 10/20/21 – 12/20/21  32/100k pop  256,009  53.76% |  |
| **Observed Performance Metrics** |  |  |
| **Community Receptivity to CICT**  % of cases interviewed  % of interviewed cases naming contacts  Timing of specimen collection (assumed days post symptom onset) | 56%  47%  2 days |  |
| **CICT Program Performance Metrics**  % of all contacts identified^†^ % of identified contacts notified  Timing of test results notification  (reported days post specimen collection)^‡^  Timing of contact notification  (reported days post specimen collection)^§^ | 26%  57%  3 days  3 days |  |
| **Calculated CICT Effectiveness** |  |  |
| % Cases and contacts isolated**^\|\|^**  Days from infection to isolation or quarantine **^**^** | 24.33%  9 days |  |
| ^*^Mean daily reported cases for the two months starting from the beginning of the evaluation.  ^†^% of contacts identified = # of named contacts / expected # of contacts per case.  Expected # of contacts per case = # of reported cases * average # named contacts per case  ^‡^This is the reported median days from specimen collection to positive test results reported to health departments. ^§^This is the reported median days from specimen collection to contact notification.  ^¶^Including contacts who later become cases. Calculated as follows using the observed performance metrics in this table, assumed compliance with isolation and quarantine guidance among cases and contacts in Appendix Table A4, and an assumed *k*=1.2:  [(% Cases interviewed * Compliance) + *k* $*$% Contacts identified   * (% Contacts monitored * Compliance + % Contacts notified but not monitored * Compliance)] /(1+*k*)  where *k* is approximated from the effective reproduction number (*R_e_*), since undetected infected contacts will infect *R_e_* additional individuals on average.  ^**^The average length of time from infection to isolation and quarantine between cases and contacts which later became cases. We assumed a 4-day pre-symptomatic period. We further assumed that interviewed cases and notified contacts to begin isolation and quarantine the day after their interactions with the health department. For more details, please refer to: Jeon *et al.* [15] , Technical Appendix, Figure A2. | | |

| **Appendix Table A5**. Assumed proportions of confirmed cases and their contacts that effectively isolated or quarantined^*^ | |
| --- | --- |
|  | Assumed Compliance with isolation/quarantine guidance |
| **Confirmed Cases that completed case interview** | 80% |
| **Confirmed Cases that did not complete case interview**^†‡^ | 0% |
| **Contacts that are notified** | 30% |
| **Contacts that are not notified by their health department**^‡^ | 0% |
| **Notes**: Each row is a mutually exclusive group of cases or contacts. The sum of the column does not add up to 100%, as the numbers represent the assumed compliance within each group. 0% compliance means none of the cases or contacts in a group isolated or quarantined effectively. 100% means all the cases or contacts in a group isolated or quarantined effectively after being interviewed or contacted.  ^*^Based on a review of the literature. Findings and sources were as follows:  A review of multiple cross-sectional population surveys in the UK suggests that 40-45% of people who had COVID-like symptoms self-reported fully complying with isolation guidance during their infectious periods [44].  A survey in the U.S. found that 85% of respondents who had COVID-like symptoms or tested positive stayed home (according to CDC guidelines) except to get medical care [45].  And a third survey, also in the U.S., found that 93% of adults said they would definitely (73%) or probably (20%) quarantine themselves for at least 14 days if told to do so by a public health official because they had the coronavirus (*i.e.,* they were confirmed cases, not just exposed contacts) [46].  ^†^Includes cases that were not reached and those that were reached but who did not agree to be interviewed.  ^‡^Compliance was set to zero for these case/contact groups categories because any transmission reductions from quarantine and isolation are not attributable to direct interactions with the health department’s CICT staff, and therefore outside of the scope of this analysis. Their inclusion here is to help distinguish between the various cases/contacts types. | |

In addition to the transmission reduction due to the CICT program, there are also other, non-CICT, NPIs that might affect COVID-19 transmission (*e.g.,* masking, business or school closures, large gathering restrictions). We estimate their impact on transmission reduction by fitting the curve of cumulative cases modeled with the above epidemiological model to the jurisdiction’s reported cumulative cases. Essentially, what we are doing here is adding a value between 0 and 1 that multiplies each of the contact rates $\beta_{i}$. The value that minimized the deviation (*i.e.,* the transmission reduction that minimized the mean squared error) between the fitted and reported cumulative case curves is the estimate of the effectiveness of non-CICT NPIs. This fitting process gives us an estimated percentage reduction in transmission attributable to other (non-CICT) NPIs. An implication of this fitting process is that the effects of other NPIs are implicitly constant over the entire study period (two months).

### Distinguishing Masking from other Nonpharmaceutical Interventions

After completing the above fitting procedure, we simulated hypothetical epidemiological curves of what would have happened if mask-wearing would have been lower, to mimic the impact of the mask mandate. We accomplished this by first calculating the transmission reduction attributable to mask-wearing. Given the efficacy of masks measured by the percentage of virus trapped by the mask ($e$), and the percentage of the population that wear masks properly ($p$), the average chance of transmission is reduced by $\left( 1-e\cdot p \right)^{2}$ due to masks [47]. We defined this transmission reduction $\left( 1-e\cdot p \right)^{2}$ as mask effectiveness. Following the literature, we assumed three levels of mask efficacy (10%, 20%, and 30%) [11], and assumed several pre- and post-mandate mask-wearing scenarios (pre-mandate range: 36% ­– 56%; post-mandate range: 61% – 71%). We then estimated the transmission reduction due to all other NPIs (other than mask-wearing) using the following equation:

*Combined NPI effectiveness, obtained from the fitting process*

*= 1- (1- mask effectiveness) * (1- all other NPIs effectiveness)*

Then, we assumed that in the absence of the mask mandate, the percentage of the population wearing masks would have been lower (see Appendix Table A1 for all mask effectiveness scenarios we consider). Assuming the effectiveness of all other NPIs remains unchanged, we can obtain a hypothetical NPI effectiveness as follows:

*Hypothetical NPI effectiveness, without mask mandate*

*= 1-(1- mask effectiveness without mandate) * (1-all other NPIs effectiveness)*

By replacing the overall NPI effectiveness with this hypothetical value, we can generate a case count curve of what would’ve happened without the increase in masking. The difference between the case counts on the last day of the simulated curves without the mask-wearing increase and Illinois’ actual reported cases on that date were the estimated number of cases averted by the increase in mask-wearing.

### Modeling the Vaccinate-or-Test Requirement

As for the vaccinate-or-test requirement, after completing the above “fitting” procedure, we simulated hypothetical epi curves of what would have happened in the absence of an increase in vaccination. We accomplished this by reducing the size of the vaccinated population by roughly 50,000 and 100,000 (see Table 3 for more details). The difference between the case counts on the last day of the simulated curves without the vaccinate-or-test requirement and Illinois’ actual reported cases on that date were the estimated number of cases averted by the vaccinate-or-test requirement.
